# Morphological and pomological assessments of seedling-originated walnut (*Juglans regia* L.) trees to select the promising late-leafing genotypes

**DOI:** 10.1186/s12870-024-04941-9

**Published:** 2024-04-08

**Authors:** Fariba Einollahi, Ali Khadivi

**Affiliations:** Department of Horticultural Sciences, Faculty of Agriculture and Natural Resources, Arak University, Arak, 38156-8-8349 Iran

**Keywords:** Walnut, Variation, Leafing date, Breeding, Cultivar

## Abstract

**Background:**

In many parts of the world, including Iran, walnut (*Juglans regia* L.) production is limited by late-spring frosts. Therefore, the use of late-leafing walnuts in areas with late-spring frost is the most important method to improve yield. In the present study, the phenotypic diversity of 141 seedling genotypes of walnut available in the Senejan area, Arak region, Markazi province, Iran was studied based on morphological traits to obtain superior late-leafing genotypes in the cropping seasons of 2022 and 2023.

**Results:**

Based on the results of the analysis of variance, the studied genotypes showed a significant variation in terms of most of the studied morphological and pomological traits. Therefore, it is possible to choose genotypes for different values ​​of a trait. Kernel weight showed positive and significant correlations with leaf length (*r* = 0.32), leaf width (*r* = 0.33), petiole length (*r* = 0.26), terminal leaflet length (*r* = 0.34), terminal leaflet width (*r* = 0.21), nut length (*r* = 0.48), nut width (*r* = 0.73), nut weight (*r* = 0.83), kernel length (*r* = 0.64), and kernel width (*r* = 0.89). The 46 out of 141 studied genotypes were late-leafing and were analyzed separately. Among late-leafing genotypes, the length of the nut was in the range of 29.33–48.50 mm, the width of the nut was in the range of 27.51–39.89 mm, and nut weight was in the range of 8.18–16.06 g. The thickness of shell was in the range of 1.11–2.60 mm. Also, kernel length ranged from 21.97–34.84 mm, kernel width ranged from 21.10–31.09 mm, and kernel weight ranged from 3.10–7.97 g.

**Conclusions:**

Based on important and commercial traits in walnut breeding programs, such as nut weight, kernel weight, kernel percentage, kernel color, and ease of kernel removal from nuts, 15 genotypes, including no. 92, 91, 31, 38, 33, 18, 93, 3, 58, 108, 16, 70, 15, 82, and 32 were superior and could be used in walnut breeding programs in line with the introduction of new cultivars and the revival of traditional walnut orchards to commercialize them.

## Introduction

Persian walnut (*Juglans regia* L.) is one of the most important nut crops in the world, and its kernel and timber have high commercial values [1]. Its origin is in a large area of Asia, from the Balkans to China. Due to the high nutritional value of its kernels, the demand for walnuts is high in the world [2, 3]. In addition, walnut kernels have high antioxidant capacity and omega-3 fatty acids, which is why their medicinal value is also important [3, 4]. According to FAO, walnut is in the group of high priority fruits [5].

Male and female walnut flowers are bearing separately on the same tree (monoecious). Also, this plant is self-compatible in terms of pollination, but it is dichogamous, so either its male flowers open earlier or its female flowers, which is cross-pollinating accordingly [6, 7]. Due to that trait and the fact that walnuts have been propagated by seeds since ancient times, their diversity is high in terms of different traits related to tree, flower, leaf, and fruit [8].

Late-leafing, fruiting both terminally and laterally, low abscission of female flowers, suitable resistance to pests and diseases, relatively soft shell, high nut yield, plump kernel, light kernel, and at least 50% kernel percentage are the main characteristics of an ideal walnut [9–11]. Hybridization in walnuts is time-consuming and laborious, but it is necessary to plan for hybridization between parents with desired traits to introduce new high-quality cultivars. In addition, the study of existing seedling-originated populations to obtain superior genotypes with ideal traits is prerequisite [10]. The first step in evaluating and describing genetic resources is to use morphological descriptors so that superior genotypes can be selected for different growth conditions [12, 13].

Flowering is considered as an important parameter with respect to crop yield and avoid of late-spring frost [14]. The pattern of walnut flowering varies depending on genetic and environmental factors [15]. In the case of a cultivar with low chilling requirements growing in cold winter areas, the blooming happens too early because the chilling requirement is quickly satisfied. Early blooming increases the likelihood of damage by late winter or early spring frosts [16]. The expression of some flowering genes during walnut flower development during the growing season and winter dormancy has been studied [14].

In many parts of the world, including Iran, walnut production is limited by late-spring frosts so in some years, large parts of the walnut orchards suffer severe damage [17]. Also, due to climate change, late-spring frost is considered a main limiting factor for walnut production. The sustainable strategy to alleviate this challenge is to use late-leafing cultivars with desirable nut characteristics [16]. Late leafing was not yet considered in that work but in 1990, a new selection study was developed to select genotypes that would not be damaged by late spring frosts [3]. Then, late-leafing genotypes of walnut have been introduced in some areas that can be used to improve future commercial cultivars [18–20]. The use of late-leafing walnuts in areas with late-spring frost is the most important method to improve yield [21]. Late-leafing and early-harvesting genotypes with desirable nut traits are suitable for growers. The genetic diversity in native walnut populations provides good opportunities to find late-leafing genotypes with high-quality kernels. Thus, here, seedling-originated populations of walnuts were investigated to find promising late-leafing genotypes.

## Material and methods

### Plant material

The phenotypic variation of 141 seedling genotypes of walnut available in the Senejan area, Arak region, Markazi province, Iran was studied based on morphological traits to obtain superior late-leafing genotypes in the cropping seasons of 2022 and 2023. Arak region is located in the canter of Iran (34º05′30"N, 49º45′10"E, and 1708 m above sea level) with 13.80 ºC mean annual temperature and 320 mm rainfall. Initially, many mature trees originated from seed were labeled according to interviews with growers and local people. Many of the genotypes that had bacterial blight symptoms in shoot, leaf, and fruit as described by Arzani et al. [22], were eliminated from evaluation. Finally, by several visits, 141 genotypes that were healthy and had a full crop, were selected. The common orchard management, including irrigation, nutrition, and pest and disease control was regularly done. The formal identification of the samples was performed by Prof. Dr. Ali Khadivi. A voucher specimen of this material has been deposited in the publicly available herbarium of Faculty of Agriculture and Natural Resources, Arak University, Iran with deposition number of JR-2342.

### The characteristics evaluated

Phenotypic variation of the trees selected was evaluated in terms of 38 quantitative and qualitative traits (Table 1). For this purpose, visits in different stages of growth, the date of leafing of trees at the time of 50% leafing, and the date of flowering of trees (male and female flowers) at the time of 50% flowering were recorded. The date of leafing and flowering of the studied genotypes was evaluated. Since phenological traits show diversity due to differences in environmental conditions [23], the data related to these traits were adjusted based on a standard genotype. Accordingly, the earliest-leafing genotype was considered as control or reference standard and it was given the code zero and for other trees, the number of days after control or reference tree was recorded. From each studied genotype, 20 leaves were randomly collected and placed in separate envelopes in August after the full growth of the leaves and were taken to the laboratory to measure the traits.

**Table 1:** Statistical descriptive parameters for morphological traits used to study walnut genotypes

No	Trait	Unit	Min	Max	Mean	SD	CV (%)
V1	Full leafing date	Code	1	5	3.14	1.52	48.54
V2	Full male flowering date	Code	1	5	2.86	1.41	49.20
V3	Full female flowering date	Code	1	5	3.23	1.12	34.80
V4	Tree height	Code	1	5	3.11	1.33	42.64
V5	Tree growth habit	Code	1	3	1.71	0.96	56.14
V6	Tree growth vigor	Code	1	5	3.41	1.21	35.43
V7	Leaf length	mm	292	496	366.27	39.44	10.77
V8	Leaf width	mm	204.10	329.40	265.11	27.88	10.52
V9	Leaf color	Code	1	5	2.74	1.31	47.66
V10	Petiole length	mm	48.50	112.70	75.47	11.56	15.32
V11	Leaflet number	Number	5.40	8.40	7.05	0.59	8.33
V12	Terminal leaflet length	mm	120.80	277.90	169.83	22.06	12.99
V13	Terminal leaflet width	mm	65.91	194.21	95.59	15.32	16.02
V14	Terminal leaflet shape	Code	1	5	3.13	0.99	31.69
V15	Ripening date	Code	1	7	4.18	1.49	35.74
V16	Yield	Code	1	5	3.85	1.40	36.34
V17	Nut length	mm	28.27	48.50	35.68	3.50	9.80
V18	Nut width	mm	25.26	39.89	31.72	2.57	8.11
V19	Nut weight	g	6.19	16.06	11.46	2.05	17.86
V20	Nut shape	Code	1	9	2.19	2.10	95.75
V21	Shell hardness	Code	1	7	3.21	1.28	39.91
V22	Shell texture	Code	1	5	1.84	1.02	55.38
V23	Shell color	Code	1	5	2.13	1.48	69.44
V24	Shell seal	Code	1	5	1.52	1.00	66.05
V25	Shell surface serration	Code	1	5	1.43	0.92	64.34
V26	Shell retention	Code	1	5	2.05	1.26	61.22
V27	Shell cover	Code	1	5	2.15	1.29	60.09
V28	Shell thickness	mm	0.88	2.60	1.52	0.29	19.44
V29	Ease of kernel removal from nuts	Code	1	5	1.94	1.36	70.31
V30	Kernel length	mm	20.18	34.84	26.82	2.56	9.55
V31	Kernel width	mm	19.38	31.43	25.92	2.18	8.41
V32	Kernel weight	g	2.84	7.97	5.44	0.99	18.20
V33	Kernel color	Code	1	7	2.29	1.88	82.27
V34	Kernel vein	Code	1	5	1.65	1.16	70.24
V35	Kernel filled	Code	1	5	4.32	1.06	24.63
V36	Kernel plumpness	Code	1	5	3.64	1.25	34.31
V37	Kernel shriveling	Code	1	5	1.68	1.14	67.98
V38	Kernel percentage	%	34.41	59.18	47.66	4.20	8.81

The harvest date was considered when almost all the green skin was easily and completely separated from the nut. For fruit ripening date, when the nuts of first genotype were ripened, its date was recorded as zero and then the fruit ripening date of the other trees was recorded based on it so that the number of days after the control or reference tree was considered as the ripening date of other trees and they were clustered as early, moderate, late, or very late ripening [22]. After full ripening of the fruits, 50 nuts from each tree were randomly selected and placed in separate envelopes, and transported to the laboratory to measure the traits. Traits were measured 10 days after harvesting and storage at room temperature and complete drying of the nuts. The width and length of the nut and kernel as well as the thickness of the shell were measured using a digital caliper with an accuracy of 0.01. Nut and kernel weight was measured in g using a digital scale. Kernel percentage was calculated from the ratio of kernel weight to nut weight. The qualitative traits were evaluated based on the walnut descriptor [24] (Table 2).

**Table 2:** Frequency distribution for the measured qualitative morphological characteristics in the studied walnut genotypes

Trait	Frequency (no. of genotypes)				
	1	3	5	7	9
Full leafing date	Early (36)	Moderate (59)	Late (46)	-	-
Full male flowering date	Early (40)	Moderate (71)	Late (30)	-	-
Full female flowering date	Early (15)	Moderate (95)	Late (31)	-	-
Tree height	Low (27)	Moderate (79)	High (35)	-	-
Tree growth habit	Spreading (91)	Upright (50)	-	-	-
Tree growth vigor	Low (14)	Moderate (84)	High (43)	-	-
Leaf color	Light green (40)	Green (79)	Dark green (22)	-	-
Terminal leaflet shape	Wide oval (13)	Oval (106)	Elliptic (22)	-	-
Ripening date	Early (14)	Moderate (38)	Late (81)	Very late (8)	-
Yield	Low (17)	Moderate (47)	High (77)	-	-
Nut shape	Round (103)	Ovate (2)	Wide ovate (30)	Oval (2)	Wide oval (4)
Shell hardness	Paper (10)	Soft (118)	Moderate (1)	Hard (12)	-
Shell texture	Smooth (83)	Moderate (57)	High (1)	-	-
Shell color	Light (82)	Moderate (38)	Dark (21)	-	-
Shell seal	Excellent seal (108)	Slightly open (29)	Moderate (4)	-	-
Shell surface serration	Low (114)	Moderate (24)	High (3)	-	-
Shell retention	Low (77)	Moderate (54)	High (10)	-	-
Shell cover	Low (72)	Moderate (57)	High (12)	-	-
Ease of kernel removal from nuts	Easy (90)	Moderate (36)	Difficult (15)	-	-
Kernel color	Light (88)	Light amber (23)	Amber (22)	Brown (8)	
Kernel vein	Low (103)	Moderate (30)	High (8)	-	-
Kernel filled	Low (4)	Moderate (40)	High (97)	-	-
Kernel plumpness	Low (12)	Moderate (72)	High (57)	-	-
Kernel shriveling	Low (100)	Moderate (34)	High (7)	-	-

### Statistical analysis

Variance analysis was performed for all traits using SAS software (Version 9.0) [25]. Descriptive statistics, simple correlation between traits, and principal component analysis (PCA) were performed using SPSS (Version 16.0) software [26]. To calculate the coefficient of variation (CV), it was calculated by dividing the standard deviation of each trait by the mean of that trait. After standardizing the data, cluster analysis was done using Ward’s method and Euclidean distance coefficient using PAST software [27]. A scatter plot was created using the most important components using PAST software.

## Results and discussion

### Phenotypic diversity of studied genotypes based on the measured traits

Based on the results of the analysis of variance, the studied genotypes showed a significant difference in most of the studied morphological and pomological traits, which is the reason for the existence of diversity in the studied traits. Therefore, it is possible to choose genotypes for different values ​​of a trait. The minimum, maximum, average, and CV of traits in the desired genotypes were calculated.

According to the obtained results, the highest CV was observed in nut shape (CV = 95.75%), while the lowest CV was observed in in nut width (CV = 8.11%) (Table 1). The CV in 24 out of 37 characters measured was higher than 20%, due to the differences in the morphological and pomological characteristics of the genotypes, indicating great differences among genotypes. Traits that have a high CV have a wider range of trait quantity, which provides a greater range of selection for that trait [12].

Tree height was low in 27 genotypes, medium in 79 genotypes, and high in 35 genotypes (Table 2). The 14 genotypes had low growth vigor, 84 genotypes had medium growth vigor, and 43 genotypes had high growth vigor. The shape of the terminal leaflet was wide oval in 13 genotypes, oval in 106 genotypes, and elliptic in 22 genotypes (Table 2).

Leaf length ranged from 292 to 496 mm, leaf width ranged from 204.10 to 329.40 mm, and petiole length varied from 48.50 to 112.70 mm (Table 1). Kavosi and Khadivi [28] reported leaf length in the range of 257.30 to 618.10 mm and leaf width from 193 to 406 mm. According to the obtained results, the number of leaflets was variable between 5.00 and 8.90. Kavosi and Khadivi [28] reported the number of leaflets from 5.80 to 10.20.

The length of the terminal leaflet was in the range of 120.8–277.9 mm, and the width of the terminal leaflet was in the range of 65.91–194.21 mm (Table 1). Mirmahdi and Khadivi [29] reported the length of the terminal leaflet from 89 to 240 mm and the width of the terminal leaflet from 46 to 106 mm.

Fruit traits are one of the most important traits in walnut breeding programs because they are less affected by environmental conditions and tree age [30]. The 17 genotypes had low yield, 47 genotypes had medium yield, and 77 genotypes had high yield. Nut shape was predominantly round (103 genotypes). Shell hardness was predominantly soft (118 genotypes), and the shell seal was excellent in most of genotypes (108). The color of the shell was light in 82 genotypes, semi-light in 38 genotypes, and dark in 21 genotypes. Shell seal was closed in 108 genotypes, slightly open in 29 genotypes, and open in 4 genotypes (Table 2).

The length of the nut was in the range of 28.27–48.50 mm, the width of the nut was in the range of 25.26–39.89 mm, and the thickness of the shell varied from 0.88 to 2.60 mm. Also, the weight of the nut was observed from 6.19 to 16.06 g (Table 1). Khadivi et al. [31] reported the length of nut from 29.42 to 44.23 mm, the width of nut from 25.61 to 35.41 mm, and the weight of nut from 7.53 to 16.91 g. Bernard et al. [32] reported that the range of nut length was from 25.99 to 52.69 mm, and nut face diameter was from 23.01 to 40.55 mm.

**Fig. 1:**
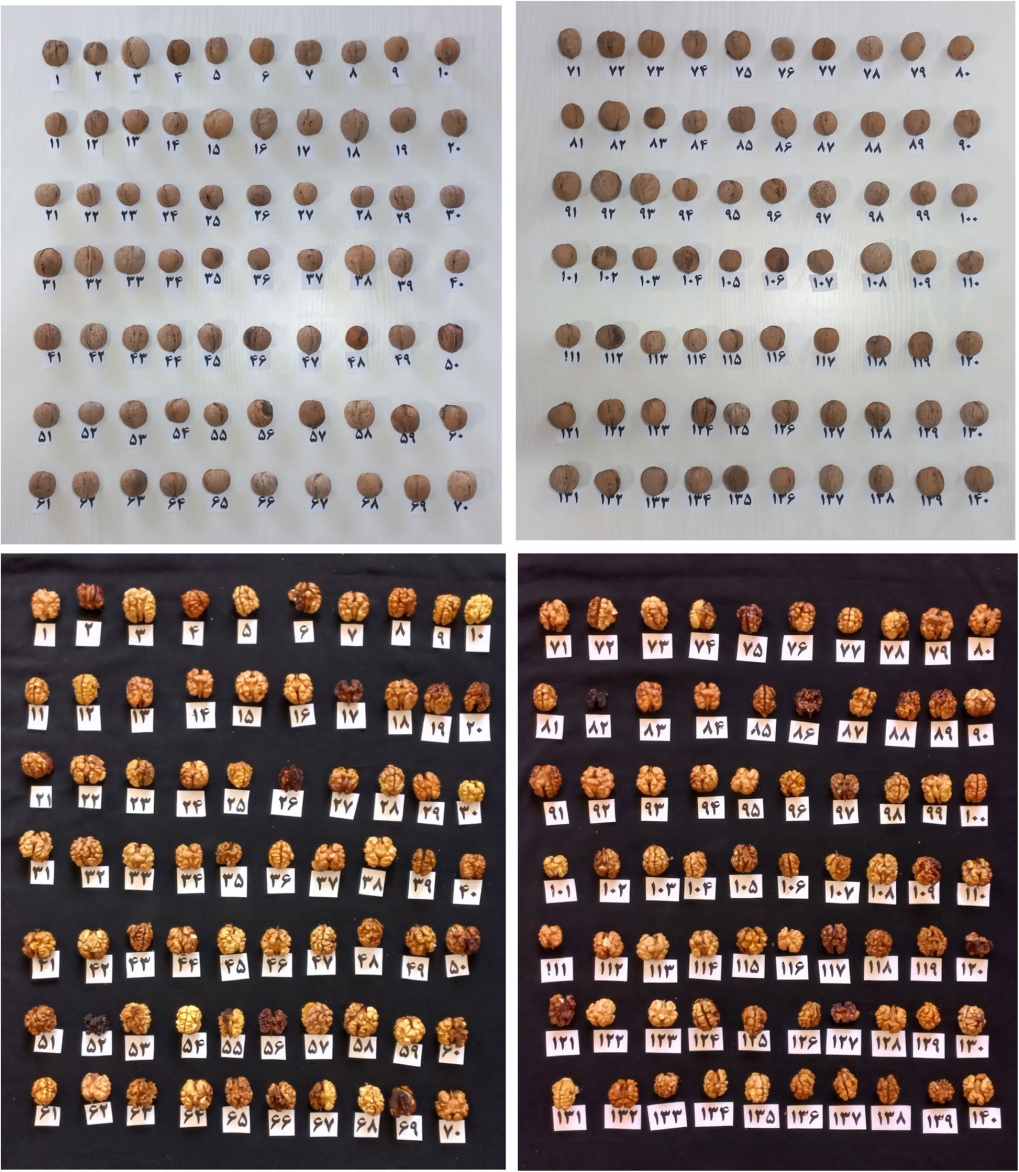
The variation of nut and kernel in terms of size, color, and shape in the walnuts studied

Kernel color was light in 88 genotypes, light amber in 23 genotypes, amber in 22 genotypes, and brown in 8 genotypes. Kernel removal from nuts was easy in 90 genotypes, medium in 36 genotypes, and hard in 15 genotypes (Table 2). The length of the kernel varied from 20.18 to 34.84 mm, the width of the kernel varied from 19.38 to 31.43 mm, the kernel weight varied from 2.84 to 7.97 g, and the kernel percentage varied from 34.41 to 59.18% (Table 1). Variation in nut and kernel of the studied walnut genotypes is shown in Fig. 1.

The analysis of the correlation coefficients showed significant positive or negative correlations between some traits (Table 3). Leaf width showed a positive correlation with leaf length (*r* = 0.63). Petiole length showed positive correlations with leaf length (*r* = 0.54) and leaf width (*r* = 0.31). The number of leaflets showed a negative and significant correlation with kernel weight (*r* = -0.23). Nut length showed positive and significant correlations with leaf width (*r* = 0.17) and terminal leaflet length (*r* = 0.20). Nut width showed positive and significant correlations with leaf length (*r* = 0.36), leaf width (*r* = 0.28), terminal leaflet length (*r* = 0.28), and nut length (*r* = 0.45). Nut weight showed positive correlations with leaf length (*r* = 0.29), leaf width (*r* = 0.23), terminal leaflet length (*r* = 0.29), nut length (*r* = 0.54), and nut width (*r* = 0.76). The thickness of the shell showed a positive and significant correlation with the weight of the nut (*r* = 0.32).

**Table 3:** Simple correlations between the quantitative morphological variables utilized in the studied walnut genotypes

Trait	V1	V2	V3	V4	V5	V6	V7	V8	V9	V10	V11	V12	V13	V14	V15	V16	V17	V18	V19	V20	V21	V22	V23	V24	V25	V26	V27	V28	V29	V30	V31	V32	V33	V34	V35	V36	V37
V1	1																																				
V2	0.72**	1																																			
V3	0.60**	0.67**	1																																		
V4	0.02	0.03	0.05	1																																	
V5	-0.05	-0.08	-0.03	0.20*	1																																
V6	-0.12	-0.03	-0.03	-0.02	-0.05	1																															
V7	0.08	-0.04	-0.02	0.08	-0.08	-0.09	1																														
V8	0.09	0.06	0.09	0.23**	-0.02	-0.06	0.63**	1																													
V9	-0.03	0.06	0.01	-0.07	0.00	0.03	0.02	-0.05	1																												
V10	0.09	0.09	-0.04	0.21*	-0.03	-0.08	0.54**	0.31**	-0.08	1																											
V11	-0.13	-0.16	-0.02	0.13	0.01	0.08	0.01	0.06	-0.06	-0.09	1																										
V12	0.10	0.03	0.07	0.16	-0.05	-0.03	0.72**	0.69**	-0.02	0.38**	-0.12	1																									
V13	-0.01	-0.07	0.04	0.05	-0.07	-0.02	0.46**	0.51**	0.03	0.12	0.04	0.64**	1																								
V14	0.05	-0.10	0.03	0.10	-0.07	-0.06	0.09	0.03	-0.03	-0.05	0.10	0.09	0.10	1																							
V15	0.13	0.17*	0.13	0.04	-0.06	-0.03	-0.03	0.02	0.23**	0.01	-0.13	0.04	0.06	0.03	1																						
V16	0.10	0.06	0.14	0.18*	-0.27**	0.09	0.01	0.08	0.07	-0.02	-0.10	0.03	0.03	0.05	-0.02	1																					
V17	0.09	0.02	-0.02	0.04	-0.01	-0.04	0.14	0.17*	-0.06	0.05	-0.01	0.20*	0.16	-0.04	0.02	-0.05	1																				
V18	0.10	-0.01	0.01	0.06	-0.03	-0.17*	0.36**	0.28**	-0.09	0.22**	-0.10	0.28**	0.16	-0.02	0.03	-0.04	0.45**	1																			
V19	0.00	-0.01	-0.06	0.03	-0.07	-0.07	0.29**	0.23**	0.04	0.16	-0.12	0.29**	0.24**	-0.17	0.02	-0.02	0.54**	0.76**	1																		
V20	0.16	0.14	0.04	0.02	-0.01	-0.04	0.02	0.03	-0.01	0.05	-0.02	-0.03	-0.04	0.14	0.05	-0.05	0.07	-0.05	-0.06	1																	
V21	-0.16*	-0.14	-0.23**	0.03	0.00	0.04	-0.04	-0.02	0.04	0.06	0.00	-0.06	0.09	-0.18*	-0.12	-0.19*	0.05	0.09	0.14	0.09	1																
V22	-0.04	0.02	-0.03	0.00	0.10	0.05	-0.12	-0.14	-0.06	-0.11	-0.12	-0.19*	-0.09	-0.11	-0.06	-0.16	0.03	-0.10	-0.07	0.11	0.10	1															
V23	0.00	0.02	0.02	-0.15	0.05	0.21*	-0.09	-0.06	0.11	-0.16	-0.02	-0.12	-0.08	-0.07	-0.13	-0.10	-0.09	-0.11	-0.06	0.02	0.19*	0.28**	1														
V24	-0.08	-0.16	-0.14	-0.07	0.13	-0.02	0.16	0.13	-0.07	0.12	-0.06	0.16*	0.10	-0.09	-0.22**	-0.20*	-0.09	-0.09	-0.05	0.07	0.19*	0.16	0.30**	1													
V25	0.09	0.07	0.03	-0.07	-0.09	0.01	0.00	-0.13	0.14	0.00	-0.12	-0.16	-0.04	0.04	-0.03	0.12	-0.07	-0.06	-0.11	0.07	0.09	0.31**	0.17*	0.22**	1												
V26	-0.12	-0.10	-0.13	-0.04	0.07	-0.04	-0.02	-0.11	0.03	0.04	0.02	-0.11	-0.16	-0.17*	-0.02	-0.20*	-0.10	-0.10	0.00	0.07	0.09	0.14	0.18*	0.29**	0.06	1											
V27	-0.14	-0.15	-0.23**	0.03	0.16	-0.03	0.00	0.03	0.07	0.05	0.11	-0.02	-0.02	-0.08	-0.09	-0.19*	0.06	0.05	0.08	0.14	0.15	0.10	0.04	0.21**	0.03	0.48**	1										
V28	-0.02	-0.07	-0.02	0.20*	-0.09	0.02	-0.03	-0.07	0.00	0.13	0.02	0.03	0.07	0.00	-0.01	0.08	0.07	0.14	0.32**	-0.11	0.03	-0.03	-0.02	-0.06	0.01	-0.09	-0.04	1									
V29	0.05	0.10	-0.08	-0.10	-0.04	-0.08	-0.12	-0.19*	0.01	-0.06	0.00	-0.15	-0.15	-0.15	-0.09	-0.20*	-0.05	-0.14	-0.10	-0.06	0.27**	0.29**	0.24**	0.24**	0.30**	0.10	0.09	0.04	1								
V30	0.08	0.02	-0.04	0.04	-0.02	-0.04	0.18*	0.22**	-0.10	0.13	-0.04	0.23**	0.15	-0.10	0.02	-0.07	0.90**	0.56**	0.61**	0.03	0.11	0.07	-0.04	-0.02	-0.06	-0.04	0.08	0.03	-0.02	1							
V31	0.02	-0.02	-0.06	0.03	-0.01	-0.08	0.38**	0.35**	-0.10	0.26**	-0.12	0.34**	0.21*	-0.03	0.05	-0.05	0.42**	0.90**	0.72**	-0.02	0.09	-0.07	-0.10	-0.05	-0.09	-0.04	0.10	0.06	-0.15	0.58**	1						
V32	0.02	0.05	-0.06	0.00	-0.04	-0.04	0.32**	0.33**	-0.06	0.26**	-0.20*	0.34**	0.19*	-0.17*	0.02	-0.05	0.48**	0.73**	0.83**	0.00	0.15	-0.02	-0.04	-0.01	-0.14	0.05	0.10	0.03	-0.05	0.64**	0.80**	1					
V33	-0.13	0.03	0.05	-0.13	0.05	-0.03	0.06	0.04	0.03	0.04	0.02	-0.01	0.02	-0.04	0.13	-0.17	0.12	0.06	0.05	-0.10	0.03	0.07	0.18*	0.05	0.11	0.08	0.06	-0.08	0.14	0.18*	0.08	0.09	1				
V34	0.00	-0.08	-0.08	-0.05	-0.03	-0.08	0.19*	0.01	0.06	0.08	0.00	0.10	-0.02	0.06	-0.14	-0.08	0.12	0.05	0.05	0.08	0.01	0.02	0.08	0.16*	0.12	0.05	0.19*	-0.16	0.11	0.14	0.05	0.07	0.10	1			
V35	0.06	0.03	-0.02	0.03	-0.08	-0.07	0.03	0.06	-0.03	0.00	-0.03	0.01	-0.02	0.06	0.04	0.05	0.04	-0.10	0.00	-0.16	-0.09	-0.10	-0.13	-0.24**	-0.16	-0.08	-0.26**	0.09	-0.08	0.00	-0.12	-0.02	-0.10	-0.13	1		
V36	-0.04	0.03	0.03	0.08	-0.03	0.01	-0.03	0.05	0.15	0.02	-0.12	0.01	0.03	0.11	0.09	0.09	-0.17*	-0.19*	-0.06	-0.04	0.02	-0.12	-0.13	-0.20*	-0.16	-0.05	-0.16*	0.18*	-0.15	-0.22**	-0.23**	-0.14	-0.06	-0.21**	0.46**	1	
V37	-0.17*	-0.20*	-0.21*	0.01	0.10	-0.07	0.12	-0.03	0.11	0.12	0.05	0.13	0.06	0.03	-0.06	-0.07	-0.07	-0.02	0.01	0.04	0.08	-0.04	0.07	0.16	0.09	0.11	0.19*	0.08	0.13	-0.11	0.05	-0.02	-0.05	0.18*	-0.16	-0.22**	1

*, **Correlation is significant at p ≤ 0.05 and 0.01 levels, respectively

Kernel length showed positive and significant correlations with leaf length (*r* = 0.18), leaf width (*r* = 0.22), terminal leaflet length (*r* = 0.23), nut length (*r* = 0.90), nut width (*r* = 0.56), and nut weight (*r* = 0.61). Kernel weight showed positive and significant correlations with leaf length (*r* = 0.32), leaf width (*r* = 0.33), petiole length (*r* = 0.26), terminal leaflet length (*r* = 0.34), terminal leaflet width (*r* = 0.21), nut length (*r* = 0.48), nut width (*r* = 0.73), nut weight (*r* = 0.83), kernel length (*r* = 0.64), and kernel width (*r* = 0.89). Previous studies on walnuts reported a positive correlation between kernel weight and nut weight [32–35]. The positive correlation between different traits shows that improving one trait may simultaneously improve another trait. Knowledge of the relationship between nut and kernel characteristics and other tree traits can guide appropriate selection schemes for walnut breeding programs. Comparison of direct and indirect effects indicates that nut and kernel weight had interactive effects on kernel percentage, *i.e.*, the nut weight reduced the kernel percentage directly but increased the kernel percentage indirectly through its effect on kernel weight [36]. Amiri et al. [36] found that kernel weight, nut weight, shell thickness, and ease of kernel removal from nuts, were the main variables accounting for kernel percentage and that they should be considered together in breeding. Studying correlations between traits helps breeders to facilitate breeding programs since correlation studies can represent linkage between related genes or multigene effects [36].

In this research, using PCA, morphological traits were included in 14 main components, whose eigenvalues ​​higher than 1 were able to justify 72.64% of the total variance (Table 4). In total, 28.25% of the observed variance was explained by the first three components and showed that these traits have the most variation among genotypes and have the greatest effect in differentiating genotypes. The first component (PC1) accounted for 12.59% of the total variance with positive and significant correlations with nut width, nut weight, kernel length, kernel width, and kernel weight, which can be called the fruit size component. The second component (PC2) expressed 8.75% of the total variance, which showed positive and significant correlations with leaf length, leaf width, petiole length, terminal leaflet length, and terminal leaflet width. The third component (PC3) expressed 6.91% of the total variance, which showed positive and significant correlations with leafing date, male flowering date, and female flowering data. PCA is a classification method that is used to identify the most important traits in all data. This analysis can clarify the main difference between the studied genotypes and reduce the amount of data. The first component explains the largest amount of variance and the subsequent components explain the remaining changes after the first component [37].

**Table 4:** Eigenvalues of the principal component axes from the PCA of the morphological characters in the studied walnut genotypes

Trait	Component													
	1	2	3	4	5	6	7	8	9	10	11	12	13	14
Full leafing date	0.07	0.03	0.86**	0.03	-0.06	0.01	-0.12	0.02	-0.03	0.06	0.08	0.00	-0.09	0.11
Full male flowering date	0.02	-0.02	0.91**	0.02	0.02	-0.01	0.00	-0.01	-0.04	-0.03	0.09	0.06	0.03	0.00
Full female flowering date	-0.08	0.03	0.83**	-0.07	-0.17	0.03	0.06	0.02	0.03	-0.05	-0.08	-0.02	0.08	-0.03
Tree height	-0.01	0.15	0.07	-0.11	0.04	-0.08	0.01	0.81**	0.22	0.01	-0.12	-0.06	-0.08	0.09
Tree growth habit	-0.02	-0.06	-0.03	-0.05	0.08	0.07	-0.04	0.13	0.86**	-0.07	0.03	0.02	0.01	-0.03
Tree growth vigor	-0.07	0.01	-0.07	-0.08	0.03	0.10	0.75**	0.04	-0.15	-0.14	-0.07	0.00	-0.03	-0.04
Leaf length	0.18	0.84**	0.00	-0.03	0.03	0.07	-0.08	0.04	-0.05	0.16	-0.04	0.02	0.05	0.01
Leaf width	0.25	0.81**	0.11	-0.09	-0.03	-0.07	0.09	0.05	0.08	0.06	-0.07	-0.02	0.03	0.03
Leaf color	-0.05	0.01	0.03	0.03	0.06	-0.09	0.09	-0.04	0.01	0.04	0.00	0.89**	0.05	0.00
Petiole length	0.09	0.62**	0.04	0.01	0.27	0.00	-0.21	0.31	-0.15	-0.04	0.12	-0.14	0.04	-0.04
Leaflet number	-0.09	-0.01	-0.08	0.01	0.02	0.05	0.08	0.08	0.00	0.09	-0.89**	-0.04	0.05	0.02
Terminal leaflet length	0.26	0.80**	-0.01	-0.09	-0.11	-0.01	-0.03	0.01	0.02	-0.01	0.10	0.03	-0.04	-0.02
Terminal leaflet width	0.13	0.61**	-0.12	0.12	-0.27	-0.06	0.11	-0.15	-0.08	-0.20	-0.12	0.11	-0.09	0.06
Terminal leaflet shape	-0.16	0.12	-0.12	-0.16	-0.39	0.02	-0.18	0.13	-0.02	0.14	-0.13	0.01	0.16	0.55**
Ripening date	-0.01	0.00	0.17	-0.27	0.01	-0.01	-0.14	0.02	-0.13	-0.37	0.15	0.38	0.48**	0.19
Yield	-0.06	0.06	0.10	-0.30	-0.28	0.02	0.28	0.32	-0.38	0.26	0.22	0.14	-0.25	-0.18
Nut length	0.84**	0.02	0.02	-0.02	-0.08	-0.13	0.08	-0.05	0.08	0.18	-0.06	-0.04	0.02	0.15
Nut width	0.81**	0.22	0.02	-0.05	-0.06	0.21	-0.18	0.08	-0.06	-0.10	-0.01	-0.05	0.01	-0.04
Nut weight	0.87**	0.13	-0.03	0.06	0.05	0.00	-0.03	0.10	-0.09	-0.12	0.03	0.09	-0.04	-0.11
Nut shape	0.03	-0.02	0.15	0.11	0.22	0.10	0.02	-0.04	-0.01	0.01	0.06	0.01	-0.18	0.79**
Shell hardness	0.10	-0.01	-0.09	0.70**	0.08	-0.06	0.09	0.00	-0.07	-0.22	-0.15	0.01	-0.16	0.15
Shell texture	0.02	-0.25	-0.03	0.32	0.08	-0.01	0.30	0.13	0.19	0.15	0.38	-0.18	0.21	0.22
Shell color	-0.06	-0.09	0.03	0.34	0.02	0.12	0.53**	-0.10	0.27	0.09	0.03	0.16	0.12	-0.04
Shell seal	-0.11	0.29	-0.11	0.50**	0.28	0.18	0.08	-0.15	0.27	0.12	0.24	-0.21	-0.02	-0.04
Shell surface serration	-0.13	-0.01	0.05	0.54**	-0.05	0.27	0.07	0.14	-0.19	0.22	0.26	0.09	0.22	0.13
Shell retention	-0.05	-0.06	-0.09	0.07	0.81**	0.02	0.04	-0.03	0.05	0.06	0.04	-0.03	0.09	-0.02
Shell cover	0.06	-0.03	-0.15	0.07	0.73**	0.19	-0.02	0.06	0.07	0.13	-0.11	0.15	-0.03	0.16
Shell thickness	0.15	-0.08	-0.13	0.26	-0.08	-0.10	-0.04	0.55**	-0.16	-0.39	0.05	0.06	-0.01	-0.11
Ease of kernel removal from nuts	-0.08	-0.16	0.08	0.70**	0.08	0.11	-0.13	-0.03	0.01	0.19	0.01	0.05	0.18	-0.16
Kernel length	0.89**	0.08	0.03	0.00	-0.02	-0.07	0.09	-0.04	0.09	0.20	-0.01	-0.08	0.06	0.08
Kernel width	0.80**	0.33	-0.03	-0.07	0.02	0.25	-0.13	0.04	-0.05	-0.10	0.04	-0.02	0.03	-0.05
Kernel weight	0.86**	0.24	0.01	-0.01	0.11	0.05	-0.03	-0.04	-0.03	-0.01	0.12	0.00	-0.02	-0.09
Kernel color	0.05	0.02	-0.01	0.13	0.06	0.04	0.07	-0.08	0.05	0.12	-0.05	0.01	0.82**	-0.14
Kernel vein	0.10	0.05	-0.05	0.10	0.16	0.13	-0.12	-0.08	-0.12	0.68**	-0.06	0.06	0.09	0.08
Kernel filled	0.00	-0.03	-0.02	-0.10	-0.18	-0.80**	-0.17	0.07	-0.04	0.05	0.01	-0.02	-0.01	-0.13
Kernel plumpness	-0.20	0.08	-0.02	-0.05	-0.01	-0.79**	-0.01	0.07	-0.05	-0.20	0.06	0.14	-0.05	0.03
Kernel shriveling	-0.07	0.09	-0.32	0.19	-0.02	0.34	-0.29	0.13	0.15	0.19	0.08	0.34	-0.19	-0.09
Total	4.66	3.24	2.56	2.15	1.87	1.77	1.41	1.37	1.35	1.34	1.31	1.30	1.28	1.28
% of Variance	12.59	8.75	6.91	5.82	5.06	4.79	3.81	3.69	3.65	3.61	3.53	3.51	3.47	3.46
Cumulative %	12.59	21.34	28.25	34.07	39.13	43.91	47.72	51.41	55.06	58.67	62.20	65.71	69.18	72.64

^**^ Eigenvalues ≥ 0.48 are significant

**Fig. 2:**
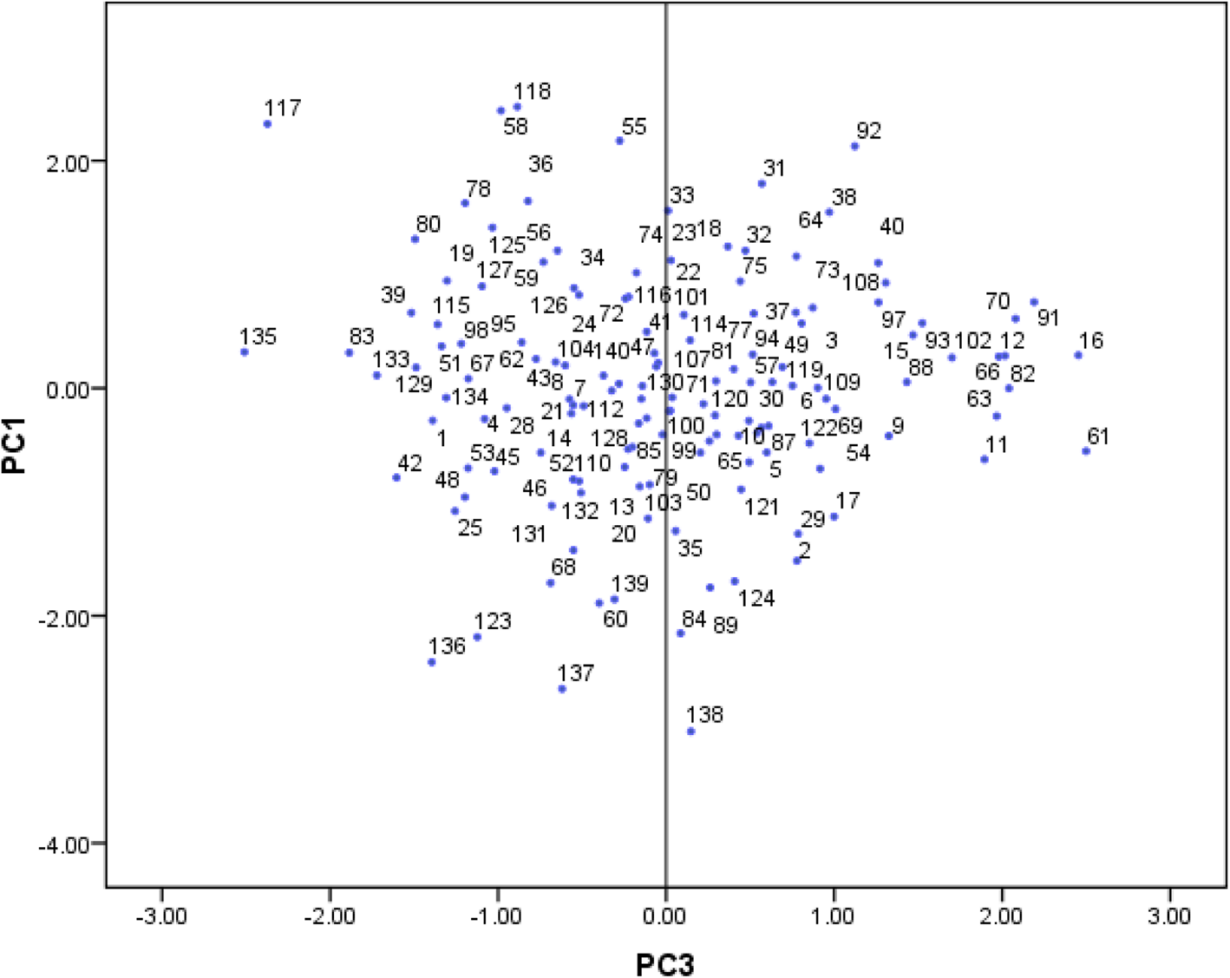
Scatter plot for the studied walnut genotypes based on PC1/PC3

The di-plot analysis method was used to display the two-dimensional distribution of genotypes. PC1 was related to nut size, PC2 to leaf size, and PC3 to phenology. Thus, the most important plot was created based on the PC1/PC3 that could help to visualize those interesting genotypes (Fig. 2). With the distribution of genotypes in the di-plot analysis, the studied genotypes were placed on the four sides of the plot in terms of phenological and pomological traits. The distribution of studied genotypes in the four directions of the plot showed that there is a great diversity between their germplasm. The genotypes of the first group were placed on the left side, and the genotypes of the second group were placed on the right side. The accumulation of genotypes in one part of the plot showed the similarity between them. Genotypes that have unique characteristics but originated from the same place were placed in one group. In this diagram, the majority of genotypes were gathered in the center of the plot.

**Fig. 3:**
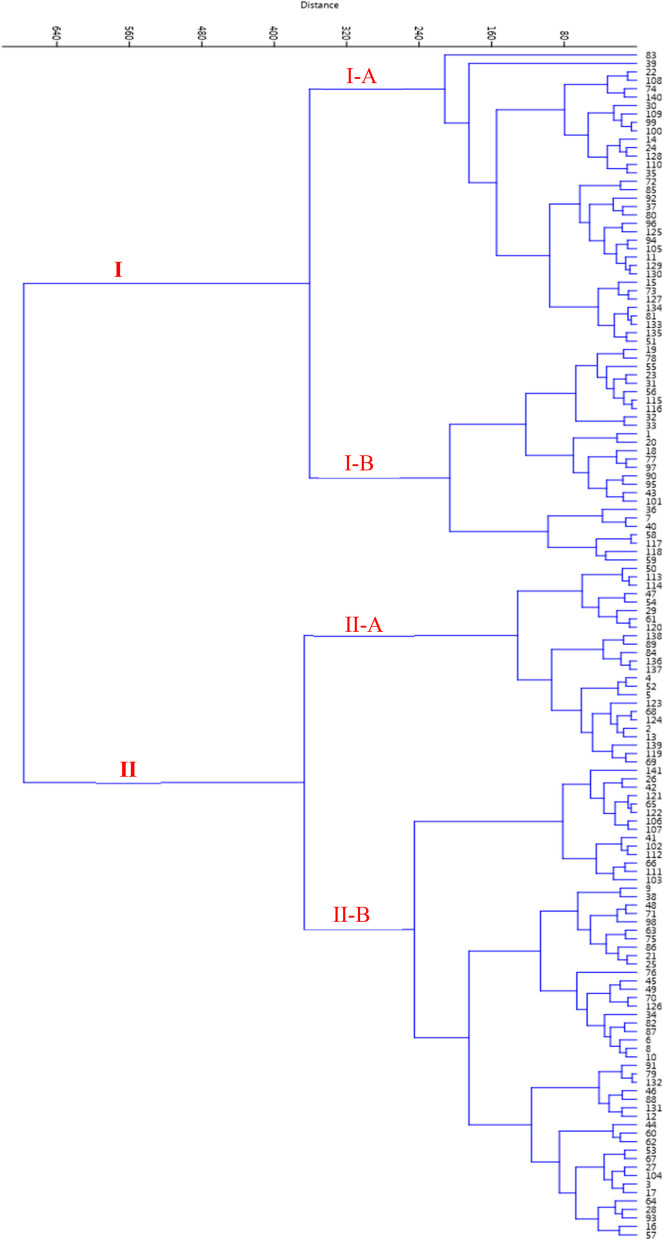
Ward cluster analysis of the studied walnut genotypes based on morphological traits using Euclidean distances

Cluster analysis is a multivariate method and has many applications in examining genetic and morphological diversity and is drawn based on the total variance. In this research, cluster analysis was done based on all measured traits studied based on Ward's method and Euclidean distance coefficient. Using the measured morphological traits, the genotypes were divided into two main groups with four subgroups (Fig. 3). Both groups were divided into two subgroups. Subgroup I-A included 35 genotypes, while subgroup I-B included 26 genotypes. Subgroup II-A included 24 genotypes, while subgroup II-B included the rest genotypes. In the research of Khadivi-Khub and Ebrahimi [37], 89 studied genotypes were divided into three main groups. In the research of Kavosi and Khadivi [28], 302 genotypes were divided into two main groups with four subgroups. Some visible differences in grouping by cluster analysis and diplot are because cluster analysis is done from all studied traits, but diplot analysis is done only using the traits of two main factors. Therefore, it is not expected that there is 100% agreement between them.

It has been stated that the high genetic variation in the walnut population is due to propagation through seeds, high heterozygosity, and dichogamy [9]. In a study [38], genotype–phenotype analysis identified 22 significant and 266 suggestive associations, some of which were for multiple traits, suggesting their correlation and a possible common genetic control. Also, genotype–environment association analysis found 115 significant and 265 suggestive SNP loci that displayed potential signals of local adaptation [38]. Vahdati et al. [39] reported that the level of gene flow in walnut populations of Kerman province, Iran was high, which meant that the high level of genetic diversity maintained within each population was less susceptible to genetic drift.

### Phenotypic diversity of late-leafing genotypes based on the measured traits

The 46 genotypes were late-leafing and were analyzed separately. According to the results of the analysis of variance, late-leafing genotypes had significant differences with each other in terms of most pomological traits, which indicates the existence of diversity in these traits. For this reason, genotypes can be selected for different values ​​of a trait. The minimum, maximum, average, and CV of pomological traits in the selected late-leafing genotypes were investigated.

According to the obtained results, the highest CV was observed in nut shape and kernel color (CV = 91.82%), while the lowest CV was observed in nut width (CV = 7.94%) (Table 5). The number of 16 out of 23 traits had a high CV higher than 20.00%, which has a wider range of the quantity of traits. Therefore, these traits are suitable for distinguishing genotypes.

**Table 5:** Statistical descriptive parameters for morphological traits used to study late-leafing walnut genotypes identified

Trait	Unit	Min	Max	Mean	SD	CV (%)
Ripening date	Code	1	7	4.39	1.26	28.61
Yield	Code	1	5	4.13	1.31	31.72
Nut length	mm	29.33	48.50	36.43	3.67	10.07
Nut width	mm	27.51	39.89	32.28	2.56	7.94
Nut weight	g	8.18	16.06	11.63	1.99	17.13
Nut shape	Code	1	9	2.74	2.52	91.82
Shell hardness	Code	1	7	3.00	1.46	48.70
Shell texture	Code	1	3	1.74	0.98	56.09
Shell color	Code	1	5	2.00	1.38	69.10
Shell seal	Code	1	5	1.39	0.91	65.18
Shell surface serration	Code	1	5	1.48	0.96	64.86
Shell retention	Code	1	5	1.70	1.13	66.65
Shell cover	Code	1	5	1.78	1.23	68.99
Shell thickness	mm	1.11	2.60	1.50	0.26	17.32
Ease of kernel removal from nuts	Code	1	5	2.04	1.45	70.83
Kernel length	mm	21.97	34.84	27.27	2.54	9.30
Kernel width	mm	21.10	31.09	26.17	2.16	8.25
Kernel weight	g	3.10	7.97	5.55	1.02	18.34
Kernel color	Code	1	7	2.09	1.92	91.82
Kernel vein	Code	1	5	1.57	1.09	69.30
Kernel filled	Code	1	5	4.35	1.12	25.75
Kernel plumpness	Code	1	5	3.57	1.24	34.76
Kernel shriveling	Code	1	3	1.30	0.73	55.85
Kernel percentage	%	37.24	59.18	47.81	4.18	8.74

The length of the nut was in the range of 29.33–48.50 mm, the width of the nut was in the range of 27.51–39.89 mm, and nut weight was in the range of 8.18–16.06 g. The thickness of shell was in the range of 1.11–2.60 mm (Table 5). Kavosi and Khadivi [28] reported a range of 5.18–15.88 g for nut weight in the studied late-leafing walnut germplasm.

**Fig. 4:**
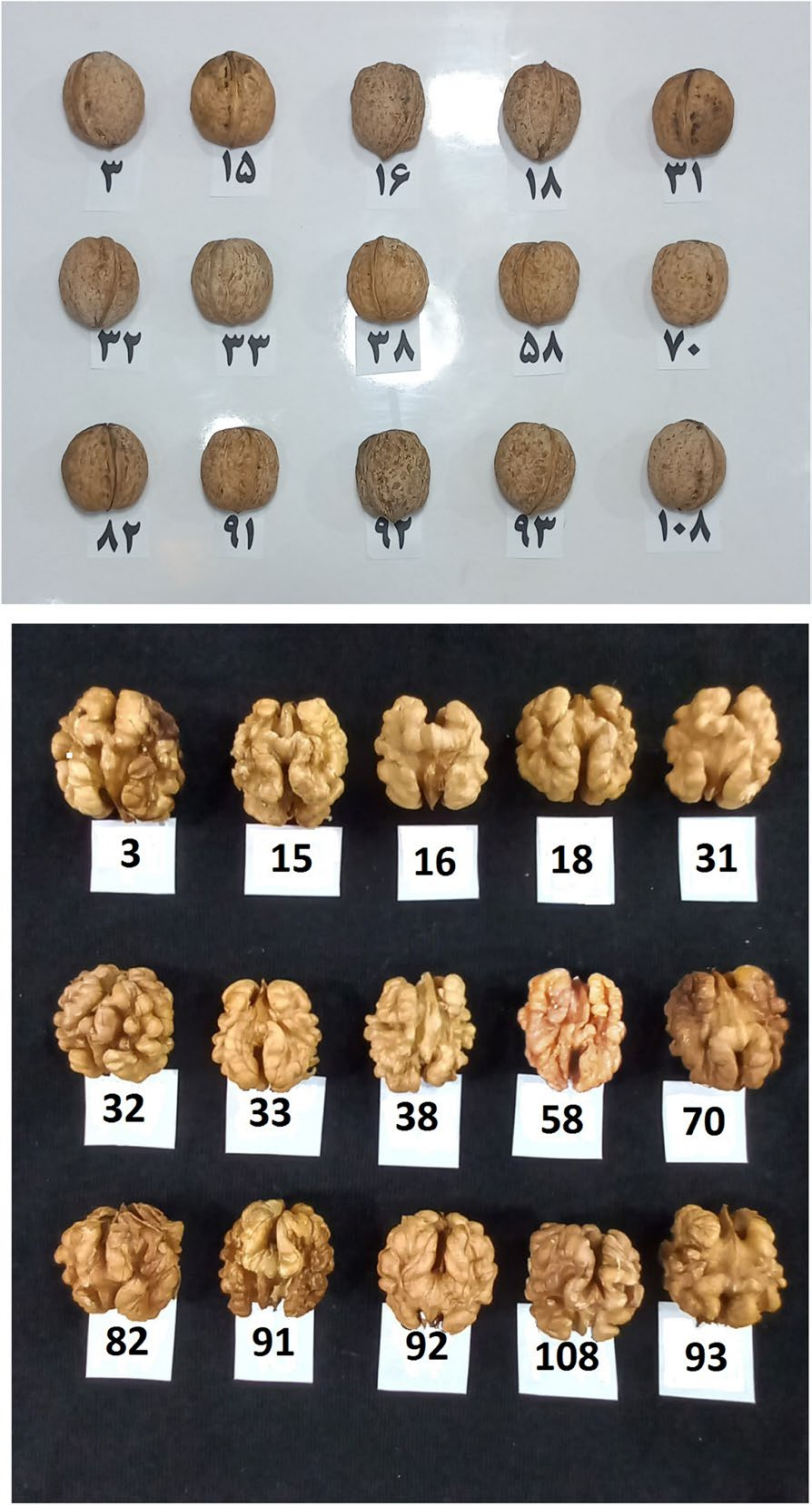
The nuts and kernels of the promising late-leafing walnut genotypes selected

Kernel length ranged from 21.97 to 34.84 mm, kernel width ranged from 21.10 to 31.09 mm, and kernel weight ranged from 3.10 to 7.97 g. Kavosi and Khadivi [28] reported a range of nut weight in their studied germplasm from 1.69 to 7.52 g. Based on important and commercial traits in walnut breeding programs, such as nut weight, kernel weight, kernel percentage, kernel color, and ease of kernel removal from nuts, 15 genotypes, including no. 92, 91, 31, 38, 33, 18, 93, 3, 58, 108, 16, 70, 15, 82, and 32 were superior and can be used by breeders to improve materials (Fig. 4).

In this research, by PCA, the traits were classified into eight main PCs, whose eigenvalues ​​higher than 1 could explain 75.54% of the total variance (Table 6). Karamatlou et al. [30] stated that decomposition into PCs reduced the 25 assessed traits to seven main components, which explained 90% of the total variance. In total, 37.59% of the observed variance was explained by the first three PCs. This indicates that these traits had the most variation among genotypes and had the most effect in differentiating between genotypes. PC1 showed 19.62% of the total variance, which showed significant correlations with nut length, nut width, nut weight, kernel length, kernel width, and kernel weight. PC2 expressed 9.81% of the total variance, which showed positive and significant correlations with yield, shell retention, shell cover, and kernel vein. The PC3 expressed 8.16% of the total variance, which showed positive and significant correlations with shell hardness, shell surface serration, and ease of kernel removal from nuts. In the research of Khadivi-Khub and Ebrahimi [37], PC1 and PC2 accounted for 21.91 and 14.39% of the total variance, respectively, and they also stated that nut and kernel-related traits were among the most important PCs for distinguishing and analyzing the materials used.

**Table 6:** Eigenvalues of the principal component axes from the PCA of the morphological characters in the late-leafing walnut genotypes identified

Trait	Component							
	1	2	3	4	5	6	7	8
Ripening date	0.05	0.09	0.03	-0.05	-0.25	0.00	0.82**	-0.11
Yield	-0.17	-0.75**	-0.11	-0.01	-0.37	-0.25	0.00	-0.13
Nut length	0.65**	0.11	0.16	-0.03	-0.06	0.44	-0.30	0.34
Nut width	0.89**	-0.09	-0.01	0.01	0.07	-0.10	-0.09	-0.10
Nut weight	0.91**	-0.01	-0.02	-0.02	0.06	-0.01	0.08	0.06
Nut shape	-0.01	0.20	-0.01	0.81**	-0.02	0.06	-0.08	0.05
Shell hardness	0.23	0.08	0.86**	0.05	0.02	-0.14	-0.03	0.00
Shell texture	0.12	-0.09	0.25	0.16	0.71**	0.15	-0.02	0.21
Shell color	0.05	0.08	0.00	-0.03	0.88**	0.02	-0.04	-0.13
Shell seal	-0.20	0.25	0.41	0.09	0.33	0.52**	0.15	-0.07
Shell surface serration	-0.18	-0.18	0.57**	0.39	0.23	0.30	0.29	0.10
Shell retention	0.02	0.71**	0.14	0.09	-0.07	0.07	0.11	-0.17
Shell cover	0.13	0.51**	0.25	0.50	-0.06	0.03	-0.17	-0.29
Shell thickness	0.10	-0.42	0.01	-0.13	0.20	-0.40	0.43	0.06
Ease of kernel removal from nuts	-0.23	0.24	0.60**	-0.17	0.23	0.25	0.17	-0.23
Kernel length	0.76**	0.14	0.17	-0.05	-0.01	0.37	-0.20	0.34
Kernel width	0.89**	0.00	-0.01	0.09	0.06	-0.15	0.01	-0.17
Kernel weight	0.91**	0.11	-0.04	-0.01	-0.01	0.06	0.10	0.07
Kernel color	-0.13	0.15	0.14	0.06	0.21	0.15	0.67**	0.27
Kernel vein	-0.07	0.68**	-0.23	0.35	-0.04	-0.13	0.20	0.27
Kernel filled	-0.02	-0.08	-0.05	-0.71**	-0.22	-0.29	-0.12	0.34
Kernel plumpness	-0.11	-0.06	0.05	-0.22	-0.13	-0.76**	-0.09	0.23
Kernel shriveling	-0.06	0.03	0.07	0.13	-0.02	0.17	-0.08	-0.80**
Total	4.51	2.26	1.88	1.87	1.86	1.79	1.71	1.50
% of Variance	19.62	9.81	8.16	8.14	8.08	7.79	7.44	6.50
Cumulative %	19.62	29.43	37.59	45.73	53.82	61.61	69.04	75.54

^**^ Eigenvalues ≥ 0.51 are significant

**Fig. 5:**
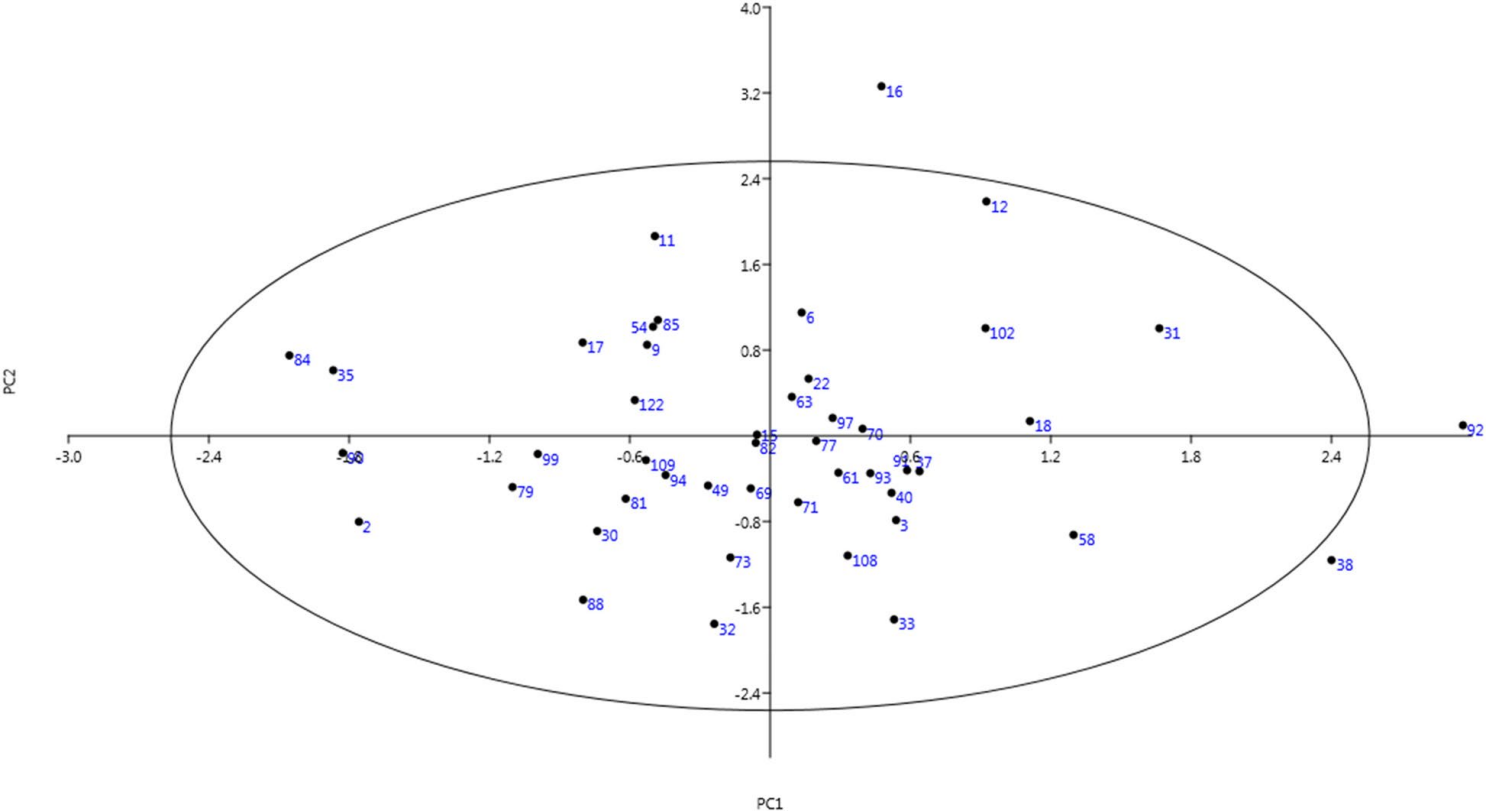
Scatter plot for the late-leafing walnut genotypes based on PC1/PC2

In this research, late-leafing genotypes were displayed in a two-dimensional plot based on the traits present in PC1 and PC2. The genotypes of the first group were placed on the left side, and the genotypes of the second group were placed on the right side. The accumulation of genotypes in an area is due to the similarity between them. Three genotypes, including no. 16, 92, and 38 were placed outside the ellipse of the plot due to having unique traits (Fig. 5).

**Fig. 6:**
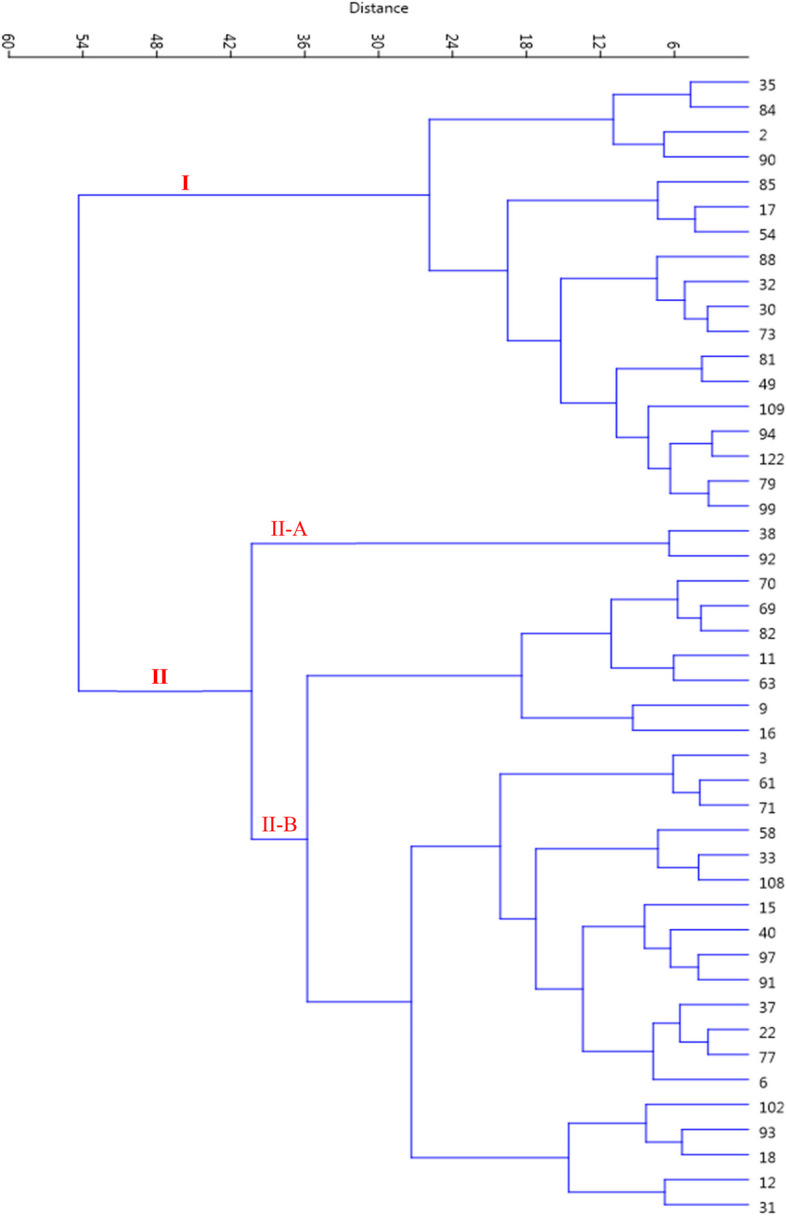
Ward cluster analysis of the late-leafing walnut genotypes based on morphological traits using Euclidean distances

Using cluster analysis, late-leafing genotypes were divided into two main groups using the measured traits. The first group included 18 genotypes (Fig. 6). The second group included the rest genotypes, forming two subgroups. In recent years, global warming has severely affected several phenological traits of trees. Early budbreak and flowering as a result of global warming increase the risk of spring frost damage, while late leafing and flowering increase the chance to escape from late-spring frost [2]. Sarikhani et al. [40] identified very late-leafing superior walnut genotypes that can be considered promising and valuable genotypes in future breeding programs.

## Conclusions

In the present study, a wide range of phenotypic variations in walnut genotypes were observed, and this variety can be used in breeding programs to improve suitable cultivars. Among the measured traits, kernel color and nut shape had the highest coefficients of variation. The results showed that there is a relatively high variety, especially in the quantitative and qualitative characteristics of walnuts, such as nut weight, nut color, and nut shape. Among many investigated traits, positive and negative correlations were observed, especially between nut and kernel-related traits. Thus, to improve cultivars and produce a suitable population, two key traits, including nut weight and kernel weight, are the main traits that should be considered in walnut breeding programs, and breeders should try to reduce shell weight and shell thickness, while increasing nut weight and kernel weight should be prioritized. In general, the grouping of genotypes using morphological and pomological traits is very useful in helping breeding programs. In general, the results of the present study provided information about the morphological and pomological characteristics of walnuts, which can be used in the protection and management of this valuable germplasm. It is suggested to use more protection of walnut genetic resources and to use this valuable germplasm in breeding programs. Based on important and commercial traits in walnut breeding programs, such as nut weight, kernel weight, kernel percentage, kernel color, and ease of kernel removal from nuts, 15 genotypes, including 92, 91, 31, 38, 33, 18, 93, 3, 58, 108, 16, 70, 15, 82, and 32 were superior and can be used by breeders to improve materials. Because at the time of blooming of these genotypes, the possibility of spring frost decreases, therefore, late-blooming genotypes can be used as parents in breeding programs. The superior genotypes introduced in this research should be used in walnut breeding programs in line with the introduction of new cultivars and the revival of traditional walnut orchards to commercialize them. The genetic diversity of other seedling walnuts available throughout the country should be evaluated and investigated. Collecting all the top genotypes and creating core collections is recommended.

## Data Availability

The data that support the findings of this study are available from the corresponding author upon reasonable request.
